# Pulsed laser deposition of single-crystalline Cu_7_In_3_/CuIn_0.8_Ga_0.2_Se_2_ core/shell nanowires

**DOI:** 10.1186/1556-276X-9-650

**Published:** 2014-12-02

**Authors:** Yu Zhao, Hui Li, Yan-Yan Zhu, Lei-Lei Guan, Yan-Li Li, Jian Sun, Zhi-Feng Ying, Jia-Da Wu, Ning Xu

**Affiliations:** 1 Department of Optical Science and Engineering, Key Laboratory for Advanced Photonic Materials and Devices, Fudan University, Shanghai 200433, People’s Republic of China; 2 Department of Physics, Shanghai Electric Power University, Shanghai 201300, People’s Republic of China

**Keywords:** Pulsed laser deposition, Nickel catalyst, CuIn_0.8_Ga_0.2_Se_2_, core/shell nanowires, Light absorption

## Abstract

Single-crystalline Cu_7_In_3_/CuIn_0.8_Ga_0.2_Se_2_ (CI/CIGS) core/shell nanowires are fabricated by pulsed laser deposition with Ni nanoparticles as catalyst. The CI/CIGS core/shell nanowires are made up of single-crystalline CI cores surrounded by single-crystalline CIGS shells. The CI/CIGS nanowires are grown at a considerably low temperature (350°C ~ 450°C) by vapor-liquid-solid mode combined with vapor-solid mode. The distribution density of the nanowires increases with the increasing of the deposition duration, and the substrate temperature determines the lengths of the nanowires. The U-V absorption spectra of the CIGS thin films with and without the CI/CIGS core/shell nanowires demonstrate that the CI/CIGS nanowires can remarkably enhance the absorption of CIGS thin films in the spectrum range of 300 to 900 nm.

**PACS:**

61.46. + w; 61.41.e; 81.15.Fg; 81.07.b

## Background

Nowadays, semiconductor nano-materials like nanowires, nanorods, and nanotubes have aroused great interest in material science and applications owing to their unique characteristics different from film or bulk materials. As an important material for the absorption layer of thin-film solar cells, Cu(In,Ga)Se_2_ (CIGS) has been one of the focuses of the research due to its high optical absorption coefficient (10^5^ cm^−1^), good radiation stability, and desirable conversion efficiency [1-6]. So far, solvothermal methods [7,8], vapor-liquid-solid techniques [9], solution-liquid-solid [10], solid-state reactions [11], and other chemical approaches [12] have been used to fabricate CuInSe_2_ (CIS) nanowires with random distribution on the substrate. The CIS nanotube arrays have also been prepared by nanotube-confined galvanic displacement method [13]. In the above methods, the fabricated CIS nanowires or nanotubes does not contain Ga element, while the Ga element is vital to increase the bandgap of CIS and improve the optical and electric properties of CIS layer [14,15]. But the syntheses of quaternary compounds that conform to the desired chemical composition have very high requirements for the equipments. Lee et al. have prepared self-assembled CIGS nano-dots and nano-ridges by ion beam etching on the surface of CIGS thin films [16]. Compared to these methods, the pulsed-laser deposition (PLD) is a more proper alternative to prepare multiple compounds. ZnSe and CdS nanoneedles have been synthesized by PLD using Ni or Au as catalyst in our previous work [17,18]. On one hand, congruent evaporation makes PLD an appropriate method for synthesis of multicomponent compounds such as CIGS thin films [19]. On the other hand, the energetic species from the instantaneous plasma produced by the pulsed laser will migrate on the substrate surface and make the low temperature of bulk substrate possible.

In this study, we have successfully synthesized single-crystalline Cu_7_In_3_/CuIn_0.8_Ga_0.2_Se_2_ (CI/CIGS) core/shell nanowires using PLD method. To our knowledge, quaternary CIGS nanowires were fabricated for the first time by PLD method. The single-crystalline CI/CIGS core/shell nanowires with semiconductor CIGS shells and metallic CI cores are desired structure for the electrodes of novel solar cells. The morphology, structure, and composition of the CI/CIGS core/shell nanowires were analyzed; their growth mechanism was discussed; and their effects on light absorption of the base CIGS thin films were also investigated.

## Methods

The CI/CIGS core/shell nanowires were deposited on soda-lime glass substrates by PLD method using Ni as catalysts. The experimental setup mainly consists of a Nd:YAG laser with a wavelength of 532 nm, a deposition chamber with rotating multitargets, and a base pressure of 10^−3^ Pa. High-purity Ni and hot-pressed CuIn_0.8_Ga_0.2_Se_2_ targets (purchased from Beijing Founde Star Science & Technology Co., Ltd.) with diameter and thickness of 1.5 and 0.5 cm, respectively, were used as evaporation sources. Prior to the deposition, substrates were ultrasonically cleaned in acetone and ethanol successively. To prepare the CI/CIGS core/shell nanowires, there were two steps involved. Firstly, Ni catalysts were deposited on the substrates by PLD with a laser pulse energy of 50 mJ and a repetition rate of 5 Hz for 10 min (without substrate heating). Secondly, the CI/CIGS core/shell nanowires were grown by PLD on Ni nanoparticle-covered glass substrates at different substrate temperatures from 300°C to 500°C and for different durations varying from 5 to 60 min. The laser pulse energy and repetition rate were set at 50 mJ and 10 Hz, respectively.

The surface morphology of all the samples was examined by a field emission scanning electron microscopy (FESEM; XL30FEG, Philips, Amsterdam, Netherlands). The micro-morphology of a single CI/CIGS core/shell nanowire prepared at the substrate temperature of 400°C for a duration of 30 min were examined by a transmission electron microscopy (TEM; JEM-2100F, JEOL, Akishima-shi, Japan). The crystalline structures of the CI/CIGS core/shell nanowire were further characterized by a high-resolution transmission electron microscopy (HRTEM) and fast Fourier transform (FFT). The composition of the CI/CIGS core/shell nanowire was analyzed by an energy-dispersive spectroscopy (EDS) fitted on the TEM. The crystalline structures of the CI/CIGS core/shell nanowires with the base CIGS thin films were also analyzed by an X-ray diffraction (XRD; D/MAX-IIA, Rigaku, Shibuya-ku, Japan) with CuKa X-rays. The UV-vis absorption spectra of the CIGS thin films with and without the CI/CIGS core/shell nanowires were detected by an ultraviolet-visible spectrophotometer (U-3000, Hitachi, Chiyoda-ku, Japan).

## Results and discussion

**Figure 1:**
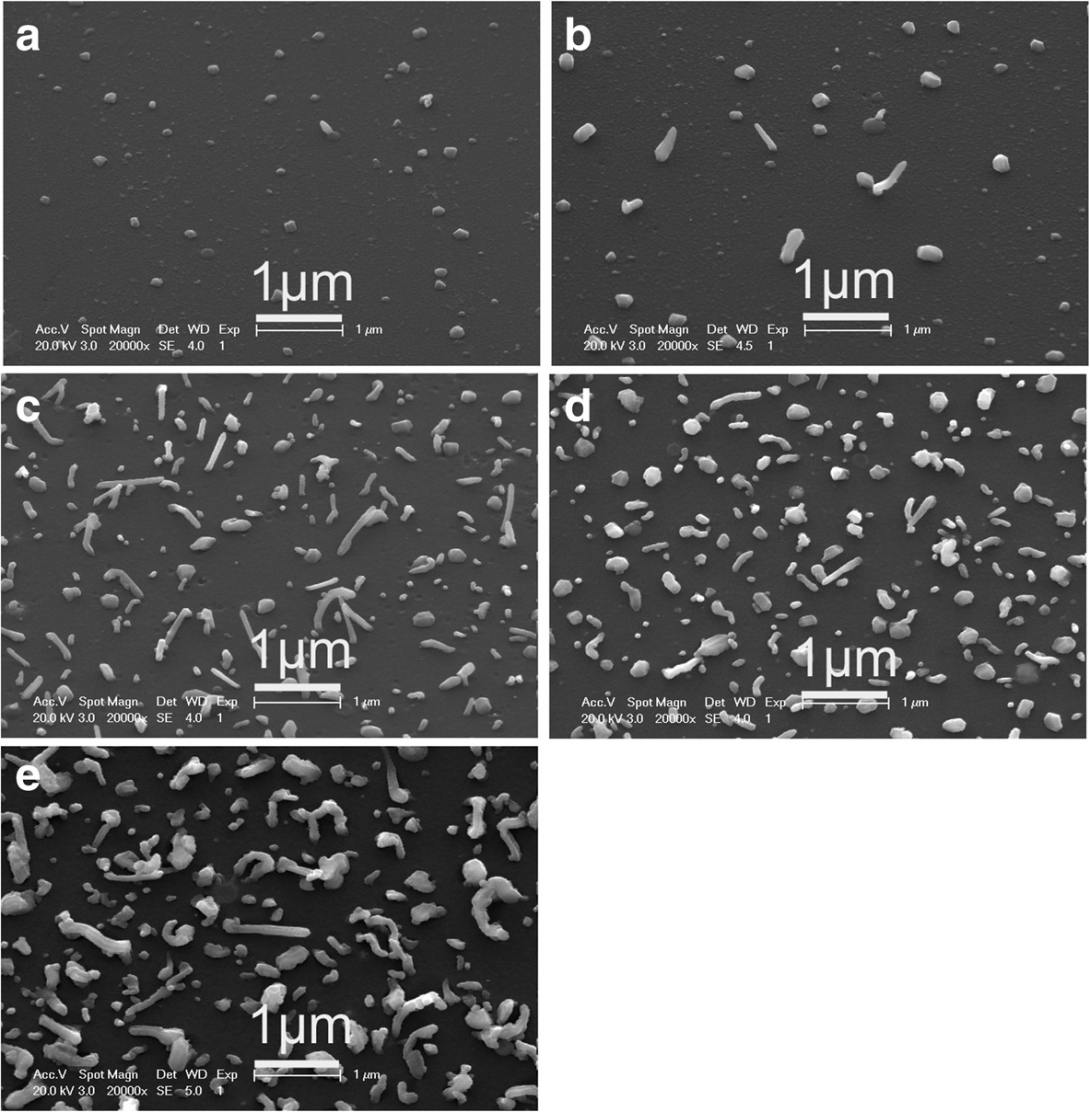
**Surface SEM images of the CI/CIGS core/shell nanowires grown at different deposition durations.** The deposition durations are **(a)** 5, **(b)** 10, **(c)** 20, **(d)** 30, and **(e)** 60 min, respectively. The substrate temperature of all the samples is 400°C.

**Figure 2:**
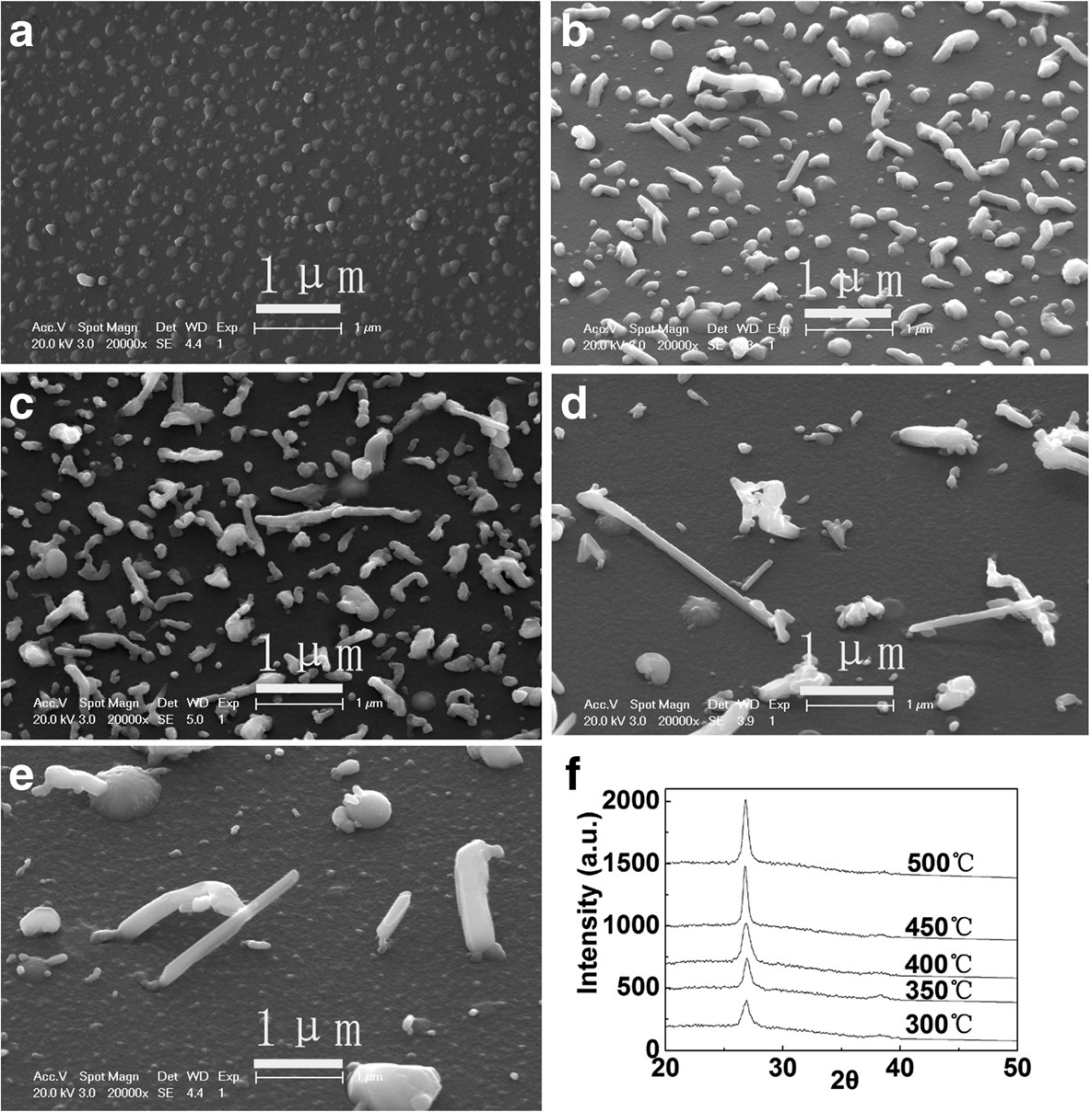
**Surface SEM images of the CI/CIGS core/shell nanowires grown at different substrate temperatures.** The substrate temperatures are **(a)** 300°C, **(b)** 350°C, **(c)** 400°C, **(d)** 450°C, and **(e)** 500°C. **(f)** The XRD of the sample deposited at the 400°C substrate temperature. All the deposition duration was 30 min.

In order to understand the growth process of the CI/CIGS core/shell nanowires, the deposition duration was changed in a series of experiments. In the deposition of the CI/CIGS core/shell nanowires, the deposition duration was set as 5, 10, 20, 30, and 60 min, respectively, while the substrate temperature of CIGS deposition (400°C) were kept unchanged. The influence of deposition duration on growth of the CIGS nanowires is shown in Figure 1. It could be seen from Figure 1 that the distribution density of the nanowires increases with the deposition duration increasing from 5 to 60 min. However, when the deposition duration is 60 min, most of the nanowires are severely bent. If preparing straight and dense nanowires, the proper deposition duration should be between 20 and 30 min (see Figure 1b,c). Besides, the effects of the substrate temperature on the growth of the CIGS nanowires were also examined. When the substrate temperature increases from 300°C to 400°C by a step of 50°C and kept other conditions (a laser pulse energy of 50 mJ, a repetition rate of 10 Hz, a deposition duration of 60 min) unchanged, the distribution density of nanowires increases from 0 to about 6 × 10^8^ cm^−2^; after that, it declines rapidly. The average length of the nanowires increases as the substrate temperature increases from 350°C to 450°C. The longest nanowires are about 2.8 μm at the substrate temperature of 450°C. When the substrate temperature increases to 500°C, the lengths of the nanowires no longer increase while their diameters become larger (Figure 2e). The substrate temperature determines the lengths of the nanowires for the deposition duration in the range of 20 to 60 min, and the more deposition duration can only let the grown nanowires more twisted. XRD spectra were measured to characterize the crystalline structure of the as-grown nanowires containing the base CIGS thin films. Figure 2f shows a typical XRD pattern of the sample deposited at the 400°C substrate temperature. The XRD exhibits a strong diffraction peak of chalcopyrite CIGS at 2θ of 26.8°, indicating the [112] growth orientation.

**Figure 3:**
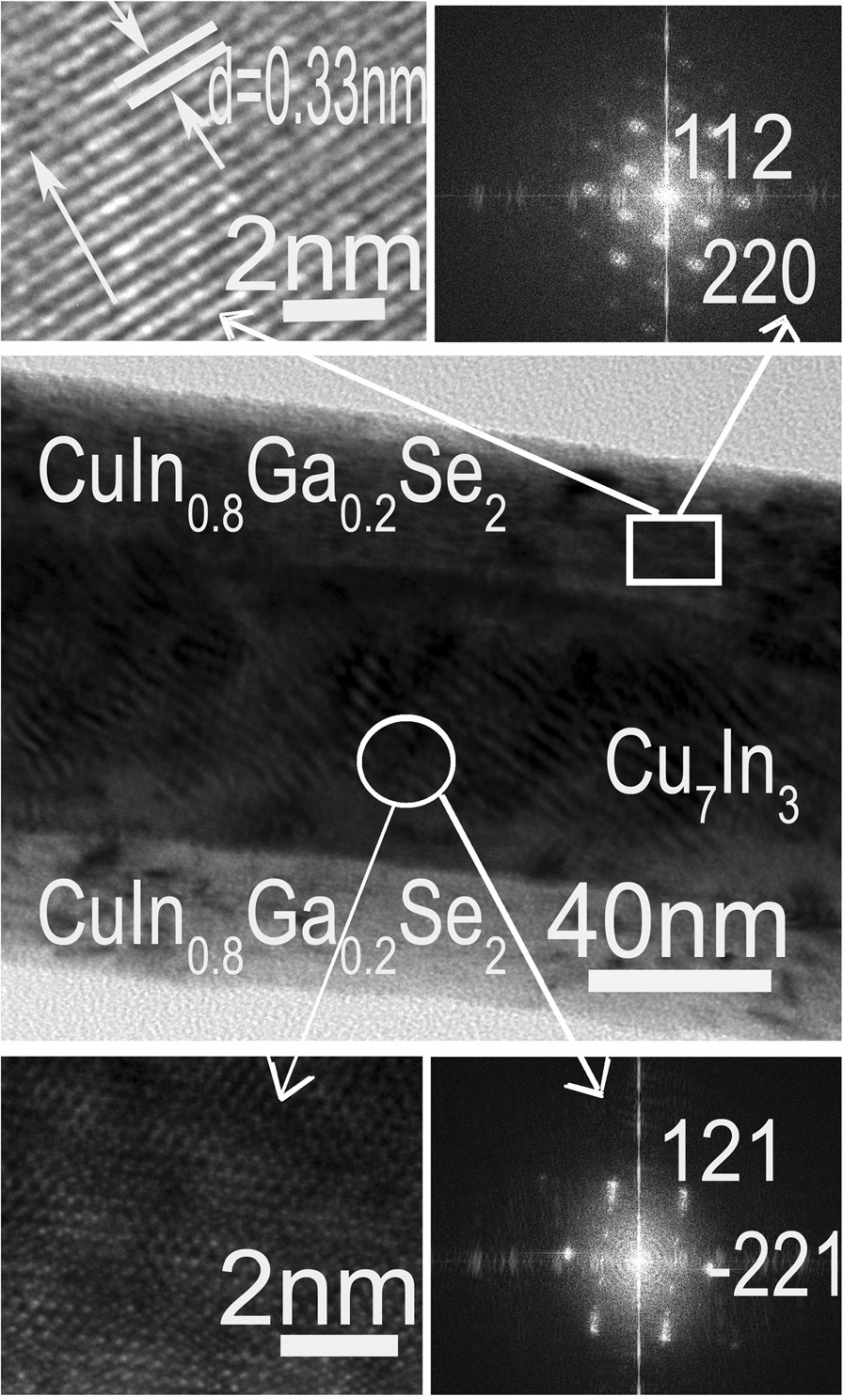
**TEM morphologies, HRTEM images, and FFT patterns of a single CI/CIGS core/shell nanowire.** The upper square-marked position is at the shell of the nanowire, and the circle-marked position is at the core of the nanowire. The sample was prepared at 400°C substrate temperature for 30 min.

**Figure 4:**
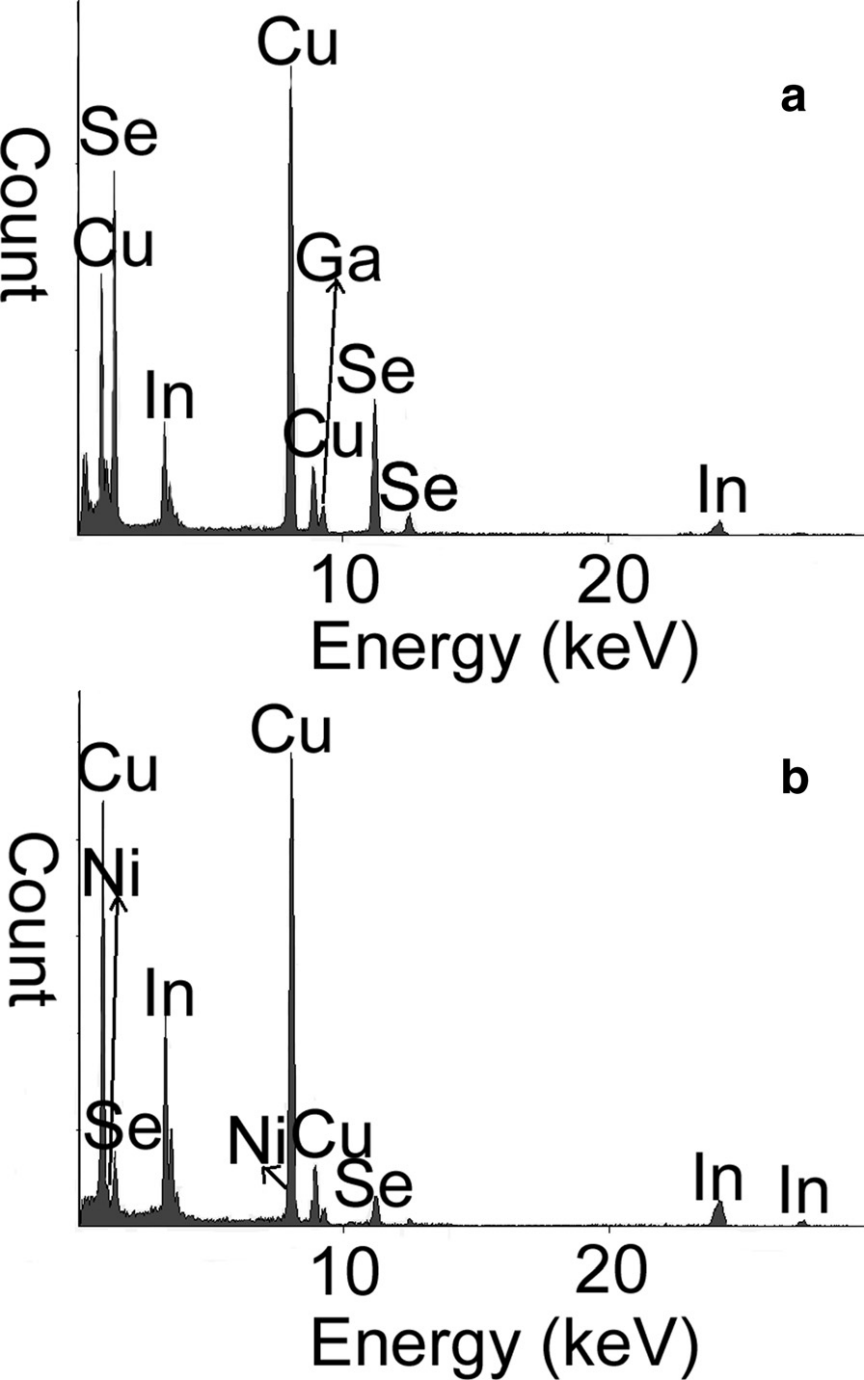
**EDS spectra at (a) square-marked and (b) circle-marked positions in Figure**3**.**

In order to further confirm the growth of the CI/CIGS core/shell nanowires, TEM, HRTEM, and EDS were carried out to analyze the morphology, structure, and composition of a single CI/CIGS core/shell nanowire. Detailed morphology of the CI/CIGS core/shell nanowire grown at substrate temperature of 400°C (as shown in Figure 1c) was clarified by the TEM (Figure 3). The nanowire is 1.5 μm in length and 100 nm in average diameter. Interestingly, the structure of the as-deposited nanowire is similar to that of a coaxial cable. There is an apparent core (dark) surrounded by a shell (light). The HRTEM image and its FFT pattern at the square-marked position exhibit that the shell of the nanowire is single-crystalline CIGS with the [112] growth orientation [JCPDS file: 35-1102], and the space of the (112) planes is about 0.33 nm (as shown in the upper-left subgraph of Figure 3). The above results are well consistent with those of the XRD. The HRTEM image and its FFT pattern in the circle-marked position exhibit that the core of the nanowire is of single-crystalline Cu_7_In_3_ structure containing few defects [JCPDS file: 65-2249]. Figure 4a,b gives the EDS spectra at the square-marked and circle-marked positions of the CI/CIGS core/shell nanowire (as shown in Figure 3). Table 1 lists the analytical results of the EDS spectra in Figure 4. From Table 1, it could be found that the element ratios of In, Ga, and Se in the CIGS shell are approximately consistent with those of the CIGS target. Because the detection of Cu element is inevitably interfered by the copper grid holding the nanowires, the detected Cu content is much larger than the real content in the nanowire. Besides, Ni element was not detected in the shell of the nanowire. So, it is inferred that the shell of the CI/CIGS core/shell nanowire should be sole CuIn_0.8_Ga_0.2_Se_2_ phase. From Table 1, it could be also seen that the core of the nanowire is mainly composed of Cu and In elements, which accords with the HRTEM and FFT results. The detected Ga and Se elements in the core may come from the shell on the EDS measurement. Moreover, the catalyst Ni element was detected in the core of the nanowire although its atomic percentage is very low.

**Table 1:** Element atomic ratios of the core and shell of the CI/CIGS core/shell nanowire

	**CIGS target**	**Sheath of CIGS nanowire**	**Core of CIGS nanowire**
In/(In + Ga)	0.8	0.79	0.91
Se/(In + Ga)	2.00	2.01	0.28
Cu/(In + Ga)	1.00	4.49	3.36
Ni/CIGS	None	None	0.0017

The data come from the analytical results of the EDS spectra in Figure 4.

**Figure 5:**
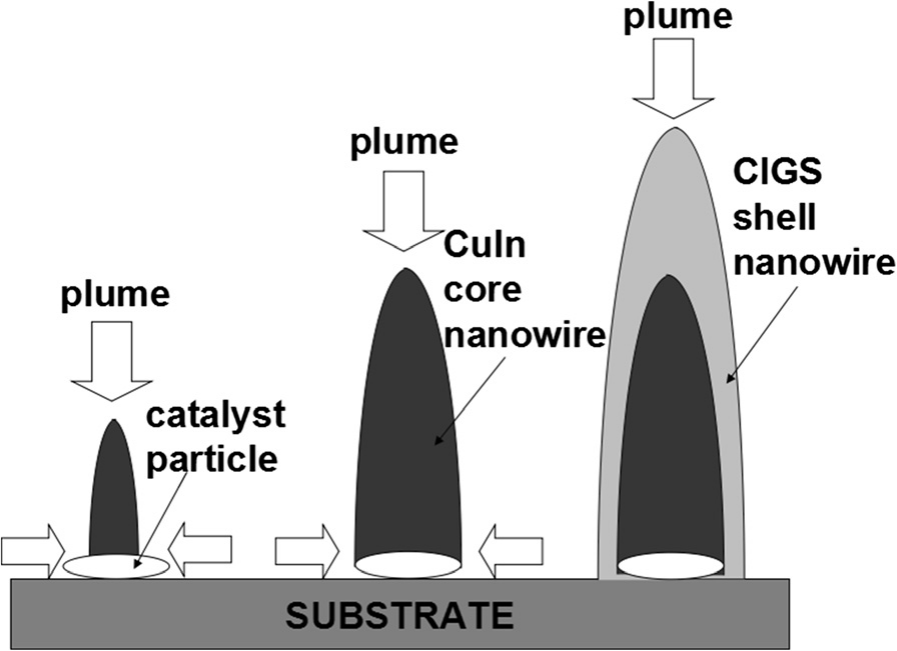
**Schematic of the CI/CIGS core/shell nanowire growth.** Vapor-liquid-solid mode for the CI core growth and vapor-solid mode for the CIGS shell growth.

**Figure 6:**
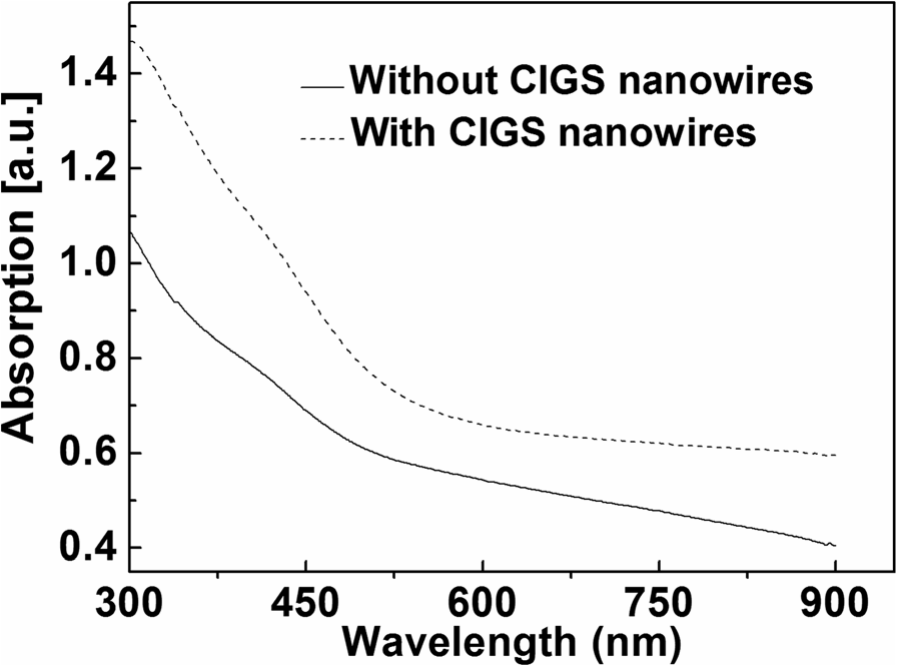
**UV-vis absorption spectra of the CIGS thin films with and without CI/CIGS core/shell nanowires.** Both the samples were prepared for the same CIGS deposition duration.

The CI core and CIGS shell of the CI/CIGS core/shell nanowires is suggested to be grown by vapor-liquid-solid (VLS) and vapor-solid (VS) mode, respectively (see Figure 5). Prior to the deposition of CIGS by PLD, a catalyst-coated substrate was heated to a preset temperature. If the substrate temperature is higher than the melt point of the Ni catalyst layer (much lower than that of bulk Ni), the Ni layer will melt and split into small liquid pellets (see Figure 5a). At the initial stage of CIGS deposition, the precursors like Cu, In, Ga, and Se atoms are directly deposited on the Ni pellets or migrate to them from the nearby places. The Cu and In atoms first diffuse into the melted Ni pellets. When the supersaturated conditions are satisfied, the Cu_7_In_3_ nanowires are grown on the melted Ni pellets (see Figure 5b). Because the CI core contains Ni element and the nanowire length is limited by the substrate temperature, the growth mode of CI core is supposed to be the VLS one under the Ni catalysis [20]. As the core nanowire grows long, the temperature of its upper part will drop and its top will solidify, which results in the cessation of the growth according to the VLS mode. Then the precursors are continuously deposited onto the grown CI core nanowire to form the shell of the nanowire without the Ni catalysis (see Figure 5c). Because Ni-catalyst is not present in the CIGS shell or on the top of the nanowire, the CIGS shell should be grown by the VS mode rather than the VLS one [20,21]. In the PLD growth of the CI/CIGS core/shell nanowires, the substrate temperature is much lower than those of the general VLS-methods [9,21,22]. For example, the ambient temperature of about 800°C to 1,000°C is required for the syntheses of GaAs and CdS nanowires by using the laser-assisted catalytic growth method (which exploits laser ablation to generate nanometer diameter catalytic clusters that are carried in the gas flow to grow the crystalline nanowires under high ambient temperature by the VLS mechanism) [22]. The considerably low temperature (350°C ~ 450°C) in the PLD growth of the CI/CIGS core/shell nanowires will be beneficial for the fabrication of the devices based on the CIGS semiconductor nanowires (usually requiring low temperature processes).It has been known that the front surface of 200-μm thick silicon wafers must be textured in order to enhance the light absorption in silicon solar cell production. However, it is difficult for 1- to 2-μm thick CIGS absorption layers in thin film solar cells to be textured. The CI/CIGS core/shell nanowires based on the simultaneously deposited CIGS thin films have the potential to be applied in the CIGS-based thin film solar cells as the absorption layers. Therefore, the effects of the CI/CIGS core/shell nanowires on the light absorption of the base CIGS thin films were investigated. The experimental conditions for the deposition of the CIGS thin films with and without the CI/CIGS core/shell nanowires grown on them were the same except that the Ni catalyst was pre-deposited for 10 min in preparation of the samples with the CI/CIGS core/shell nanowires. In order to compensate the impact of Ni catalyst, a Ni layer were post-deposited on the CIGS thin films without the CI/CIGS core/shell nanowires prepared by PLD for 10 min. Figure 6 is the comparison of the light absorption spectra of the CIGS thin films with and without the CI/CIGS core/shell nanowires. It can be seen from Figure 6 that the CI/CIGS core/shell nanowires remarkably enhance the absorption in the range of 300 to 900 nm. The absorption is increased by 33% on average. The absorption enhancement induced by the CI/CIGS core/shell nanowires will help the CIGS absorption layers capture the more incident light and increase the short circuit currents of thin film solar cells. Simultaneously, the absorption enhancement could permit a considerable reduction in the physical thickness of absorber layers in thin film solar cells and decrease the recombination of the minority carriers. Moreover, the conductor/semiconductor structure of the CuIn/CIGS core/shell nanowires is possible to form Schottky junction for the fabrication of micro solar cells, which may yield some new ideas in the design of thin film solar cells.

## Conclusions

The CuIn/CIGS core/shell nanowires were fabricated by the PLD with nickel as catalyst. The effects of the deposition duration and substrate temperature on the growth of the nanowires were examined. The SEM results indicate that the distribution density of the nanowires increases with the increase of the deposition duration, and the substrate temperature determines the lengths of the nanowires. The TEM, HRTEM, and FFT results show that the CuIn/CIGS core/shell nanowire has the structure of the defectively single-crystalline Cu_7_In_3_ core surrounded by the perfectly single-crystalline CIGS shell. The EDS results confirm the composition of the CuIn/CIGS core/shell nanowire. The CuIn/CIGS core/shell nanowires were grown at a considerably low substrate temperature (350°C ~ 450°C) by the VLS mode combined with the VL mode. The U-V absorption spectra of the CIGS thin films with and without the CuIn/CIGS core/shell nanowires indicate that the CuIn/CIGS core/shell nanowires can help the CIGS absorption layer capture more incident light and are potential to reduce the physical thickness of the absorber layers in thin film solar cells. The CuIn/CIGS core/shell nanowires are also expected to form Schottky junction for the fabrication of micro solar cells.

## Abbreviations

CIGS: CuIn_0.8_Ga_0.2_Se_2_; EDS: Energy-dispersive spectroscopy; FESEM: Field emission scanning electron microscopy; FFT: fast Fourier transform; HRTEM: High-resolution transmission electron microscopy; PLD: Pulsed laser deposition; SEM: Scanning electron microscopy; TEM: Transmission electron microscopy; VLS: Vapor-liquid-solid; VS: Vapor-solid; XRD: X-ray diffraction.

## Competing interests

The authors declare that they have no competing interests.

## Authors’ contributions

YZ designed and carried out the experiments and wrote the paper. HL, YZ, LG, and YL participated in the experiments. JS, FY, and JW participated in the design and the discussion of this study. NX conceived and designed the experiments and revised the paper. All authors read and approved the final manuscript.
